# THE RELATIONSHIP BETWEEN PAIN AND STRESS IN OLDER BLACKS

**DOI:** 10.1093/geroni/igz038.274

**Published:** 2019-11-08

**Authors:** Janiece L Taylor, Janiece L Taylor, Lauren Parker, Roland J Thorpe Jr., Keith E Whitfield

**Affiliations:** 1 Johns Hopkins School of Nursing, Baltimore, Maryland, United States; 2 Johns Hopkins Bloomberg School of Public Health, Baltimore, Maryland, United States; 3 Wayne State University, Detroit, Michigan, United States

## Abstract

Older Blacks have higher rates of undermanaged and undertreated pain than other racial/ethnic groups. Pain can lead to disability and poor quality of life. It is essential that we identify modifiable factors related to pain in this population. This study examined if stress was associated with pain among older Blacks. Data were taken from the Baltimore Study on Black Aging, (N=602, mean age 69 [SD= 9.76]). A total of 78% of participants reported bodily pain in the past month. Women had an increased odds of reporting bodily pain (OR 1.89, 95% CI 1.17, 3.07) compared to men. Using logistic regression controlling for age, self-rated health, sex, depressive symptoms, and chronic conditions, higher levels of stress were associated with increased odds of bodily pain (OR 1.04, 95% CI 1.00, 1.07). Identification of effective coping mechanisms to combat stress may lead to pain relief among older Blacks, particularly Black women.

